# Short-term disease control with alternate-day maintenance upadacitinib in refractory Hailey–Hailey disease: a case report

**DOI:** 10.3389/fimmu.2026.1935043

**Published:** 2026-09-16

**Authors:** Deping Luo, Fan Yang, Zexin Zhao, Hongxia Li, Liancheng Guan

**Affiliations:** The Second Affiliated Hospital, Guizhou University of Traditional Chinese Medicine, Guiyang, China

**Keywords:** alternate-day dosing, Hailey–Hailey disease, Janus kinase inhibitor, maintenance therapy, upadacitinib

## Abstract

Hailey–Hailey disease (HHD), also known as benign familial pemphigus, is a rare autosomal dominant genodermatosis characterized by recurrent erosive lesions in intertriginous areas. Previous case reports have described clinical improvement with upadacitinib, but post-response maintenance strategies remain poorly defined. We report a 62-year-old woman with a 7-year history of refractory HHD who showed marked improvement after 2 months of upadacitinib 15 mg once daily. After 6 months of daily treatment, the lesions had completely cleared and disease control was stable. The regimen was then changed to 15 mg every other day after discussion with the patient. The change was based on sustained clinical control, concern about the financial burden of continued daily treatment, and a preference to limit prolonged systemic exposure; it was not prompted by an adverse event. The patient adhered to the alternate-day schedule, did not resume daily treatment, and required no rescue or additional therapy. During the subsequent 3 months, no new or recurrent lesions were observed and no apparent adverse events occurred. This case therefore documents short-term disease control after transition to an alternate-day maintenance schedule. Given the relapsing and fluctuating course of HHD and the limited follow-up after dose reduction, the observation should not be interpreted as evidence of durable remission or long-term efficacy.

## Introduction

Hailey–Hailey disease (HHD), also known as benign familial pemphigus, is a rare autosomal dominant genodermatosis caused by pathogenic variants in ATP2C1, resulting in impaired calcium homeostasis and keratinocyte adhesion (1, 2). Clinically, HHD is characterized by recurrent erythema, vesicles, and erosions in intertriginous areas, often accompanied by pruritus and pain, and can substantially impair quality of life (3). Conventional therapies may provide only temporary or incomplete control in refractory disease (4). Increasing evidence suggests that inflammatory pathways contribute to disease activity in addition to the primary defect in epidermal adhesion. Type 2 cytokines, including interleukin-4 (IL-4) and interleukin-13 (IL-13), may interfere with keratinocyte calcium signaling and provide a rationale for therapies that modulate JAK–STAT signaling (5, 6). Upadacitinib has already been reported to induce clinical improvement in HHD (6–9). Wu et al. further suggested that treatment reduction might be considered in selected responders, although an alternate-day maintenance schedule was not reported (6). The present report focuses specifically on post-response management: after 6 months of daily treatment and achievement of complete clinical clearance, the patient remained clinically stable for 3 months after transition to an alternate-day regimen. Because HHD has a relapsing and fluctuating course, this finding is interpreted as short-term disease control rather than durable remission.

We present the following case in accordance with the CARE guideline.

## Case presentation

A 62-year-old Chinese woman presented with a 7-year history of infiltrated erythema, erosions, and exudation involving the bilateral axillae and inguinal regions, accompanied by pruritus, pain, and malodor. Similar eruptions had developed on the buttocks 1 year earlier (**Figures 1A–D**). Her mother and maternal uncle had experienced similar eruptions in the inguinal and axillary regions. The patient had a history of chronic urticaria for more than 10 years, which was well controlled, and denied other relevant medical history. Her first HHD-like lesions appeared at approximately 55 years of age, which is later than the usual age at onset. Late-onset HHD has been reported, including cases associated with ATP2C1 variants (10–12). ATP2C1 genetic testing was recommended to further characterize the disease, but the patient declined testing because of financial considerations; therefore, no molecular result was available. The diagnosis was based on the characteristic clinical presentation and family history together with histopathological findings and negative direct immunofluorescence.

**Figure 1:**
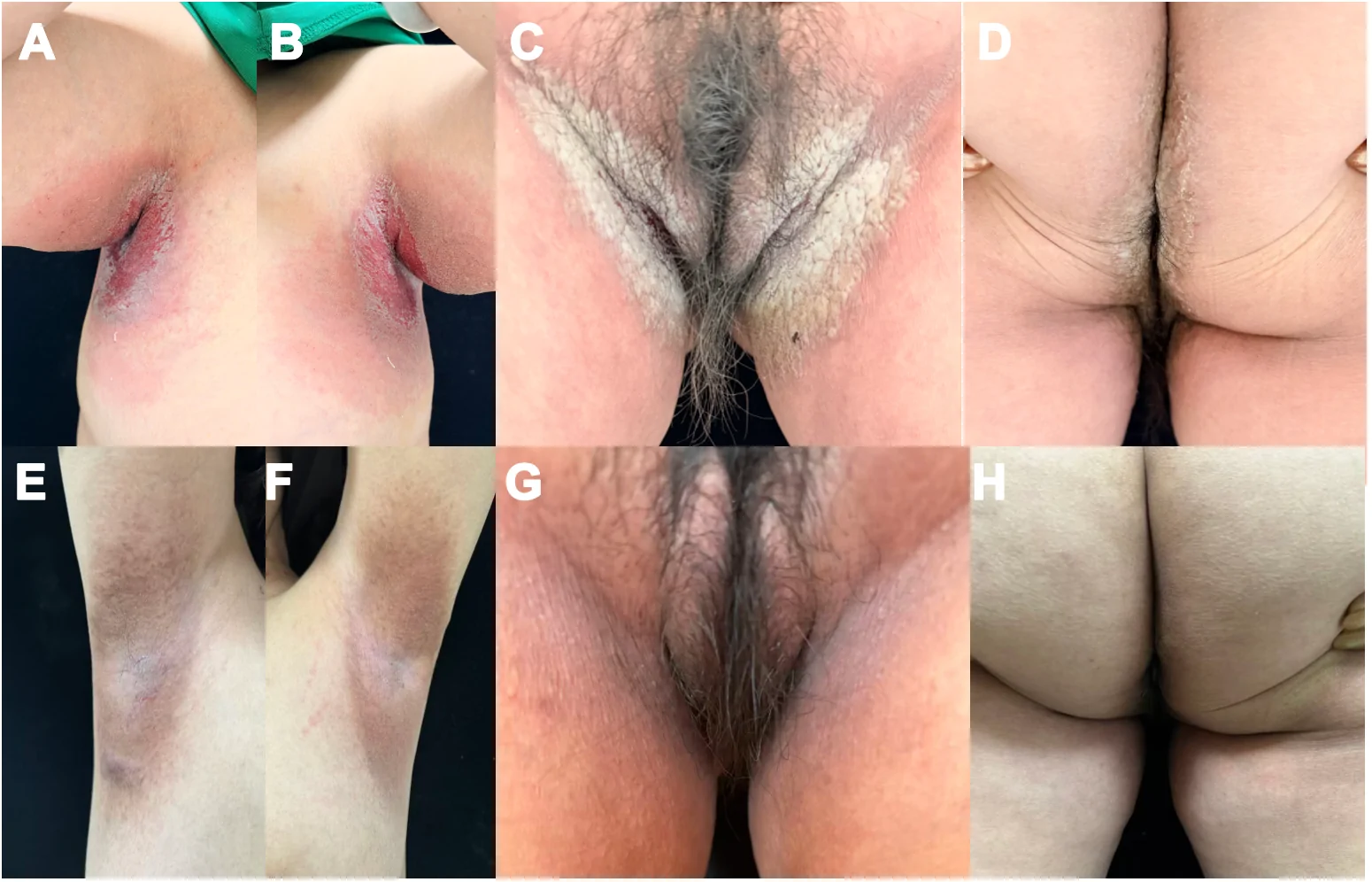
Clinical response to treatment. Before treatment, erythema, erosions, and exudation were present in both axillae **(A, B)**, with macerated plaques in the inguinal region **(C)** and perianal region **(D)**. After 8 weeks (2 months) of treatment, these lesions had markedly improved **(E–H)**, leaving only faint erythema and post-inflammatory hyperpigmentation in some areas.

Before the diagnosis was established, the patient had been repeatedly treated at other hospitals for presumed eczema with oral desloratadine citrate disodium, intramuscular compound betamethasone, and topical corticosteroids, but her symptoms did not improve. In 2022, a skin biopsy was performed from the right axilla at our hospital. Histopathological examination showed acantholytic dermatosis with dyskeratosis and incomplete acantholysis, resembling a dilapidated brick wall (**Figures 2A, B**). Direct immunofluorescence was negative for C3, IgG, IgM, IgA, and fibrinogen. After a definite diagnosis of HHD was made, the patient subsequently received oral ebastine, desloratadine citrate disodium, tripterygium glycosides, and thalidomide, as well as intralesional injection of botulinum toxin type A into the axillary lesions, topical antibiotic ointments, and topical corticosteroid ointments. However, disease control remained poor.

**Figure 2:**
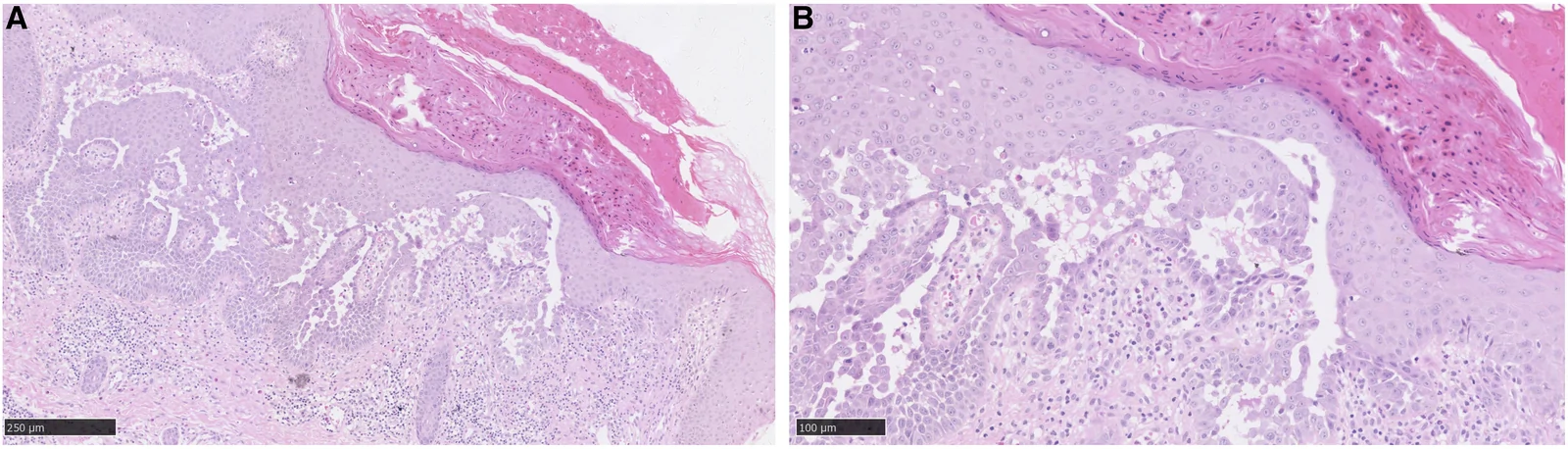
Histopathological findings. Histopathological examination of a skin biopsy specimen from the right axilla showed acantholytic dyskeratosis with incomplete acantholysis, producing a “dilapidated brick wall” appearance: **(A)** hematoxylin and eosin staining, ×40; **(B)** hematoxylin and eosin staining, ×100.

Because the patient urgently required a new therapeutic option, we decided to initiate treatment with upadacitinib 15 mg once daily. Before treatment, complete blood count, liver and renal function tests, serum electrolytes, urinalysis, immunoglobulin E, hepatitis virus markers, HIV testing, syphilis serology, an autoantibody profile, electrocardiography, chest computed tomography, tumor markers, and a T-cell-based assay for tuberculosis infection were performed, all of which showed no abnormalities.

After 2 months of treatment, the skin lesions improved markedly, with healing of the erosions and only residual pale erythematous patches and pigmentation (**Figures 1E–H**). After 6 months of daily treatment, the lesions had completely cleared and disease control was stable (**Figures 3A–D**). In the seventh month, a step-down regimen was discussed with the patient. The decision to change to upadacitinib 15 mg every other day was based on the patient’s concern about the financial burden of continued daily treatment and a preference to limit prolonged systemic exposure after sustained clinical control. The change was not prompted by an adverse event. The patient followed the alternate-day schedule consistently; daily dosing was not restarted, and no rescue or additional treatment was required. During the subsequent 3 months, the disease remained clinically stable, with no new or recurrent lesions (**Figures 3E–H**), and no apparent adverse events were observed.

**Figure 3:**
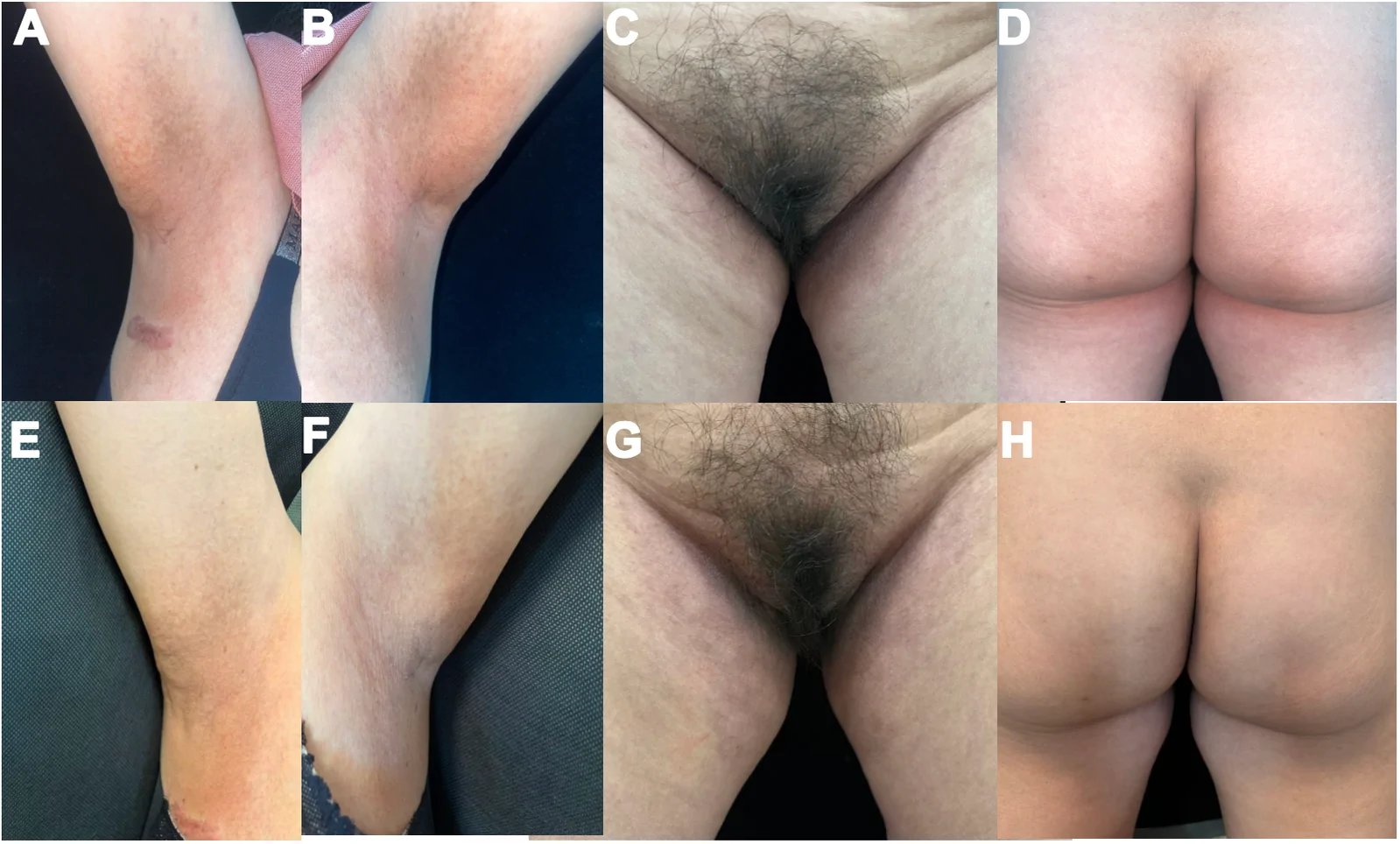
Clinical course during daily treatment and alternate-day maintenance. After 24 weeks (6 months) of daily treatment, disease control remained stable in the axillary **(A, B)**, inguinal **(C)**, and perianal **(D)** regions. After a further 12 weeks (3 months) on the alternate-day schedule, no new or recurrent lesions were observed in the corresponding regions **(E–H)**, and disease control remained clinically stable.

## Discussion

Hailey–Hailey disease is a chronic, relapsing genodermatosis caused by ATP2C1 variants and characterized by recurrent acantholytic lesions in intertriginous areas (1–3). There is no curative medical treatment, and conventional topical or systemic approaches frequently provide incomplete or temporary control in refractory disease (4, 13). Local treatment such as botulinum toxin can be useful when sweating and maceration are prominent triggers, but responses are variable and treatment failure has also been reported (14, 15). These limitations have contributed to growing interest in targeted anti-inflammatory approaches for difficult-to-control HHD.

Inflammatory signaling in HHD is unlikely to be explained by a purely type 2 model. Abnormal IL-4 and IL-13 activity and their effects on keratinocyte calcium handling support a role for type 2 inflammation (5, 6), and reported responses to dupilumab provide additional, although still case-based, support for this pathway (16, 17). At the same time, increased IL-17A-positive cells have been demonstrated in genetic acantholytic disorders including HHD (18), while clinical responses have been reported with therapies directed at IL-17A and IL-23-related pathways (19–21). These findings suggest that type 3 inflammation may also contribute to disease activity in at least some patients. Accordingly, the response observed with upadacitinib should not be attributed solely to inhibition of IL-4/IL-13 signaling. JAK1 inhibition can modify several cytokine networks relevant to cutaneous inflammation, but direct IL-17 receptor signaling is not primarily JAK–STAT dependent. The mechanistic basis of response in HHD therefore remains uncertain and is likely to be more complex than a single-cytokine explanation.

Published reports of JAK inhibition in HHD have mainly focused on induction responses, whereas post-response management has been less clearly described. Murphy et al. reported clearance during continued daily upadacitinib treatment (7). Wu et al. reported marked improvement with upadacitinib and suggested that treatment reduction might be considered in selected responders, but did not describe an alternate-day maintenance schedule (6). Other reports illustrate the uncertainty surrounding treatment de-escalation: recurrence has been described after treatment interruption, and relapse after attempted tapering has also been reported (8, 22) Against this background, the clinically relevant observation in the present case is limited but distinct: disease control remained stable for 3 months after transition from daily treatment to an alternate-day schedule following 6 months of daily therapy. The short follow-up precludes any conclusion regarding an optimal tapering strategy or long-term maintenance efficacy.

In this patient, de-escalation was undertaken only after 6 months of daily treatment, at which point complete clinical clearance and stable disease control had been achieved. No further reduction was attempted during the documented follow-up. It therefore remains unknown whether a lower treatment exposure would have been sufficient to maintain control. The relatively late onset of HHD in this patient also deserves comment. Late-onset disease has been described in association with ATP2C1 variants (11, 12), but genetic testing was not performed in the present case. Consequently, neither the late presentation nor the favorable treatment response can be linked to a specific genotype, and there is currently no basis for assuming that later age at onset predicts response to JAK inhibition.

Several limitations should be acknowledged. First, this is a single case and the follow-up after transition to the alternate-day schedule was limited to 3 months. Because HHD has a naturally relapsing and fluctuating course, a recurrence-free interval of this duration cannot establish durable remission or long-term efficacy. Second, ATP2C1 genetic testing was not available, which limits interpretation of the late-onset phenotype and precludes genotype–phenotype analysis. Third, the case cannot determine whether further treatment reduction would maintain disease control. Finally, the optimal timing and extent of de-escalation, as well as clinical or biological markers that might identify suitable patients, remain unknown. Longer follow-up and additional clinical observations are required before reduced-frequency maintenance can be generalized.

## Patient perspective

The patient expressed satisfaction with the treatment outcome, noting substantial improvement in the skin lesions and quality of life, and reported no apparent adverse effects during the documented treatment period.

## Conclusion

This case documents short-term disease control in a patient with refractory HHD after transition from daily upadacitinib treatment to an alternate-day maintenance schedule. The observation is clinically relevant because post-response maintenance strategies in HHD remain poorly defined, but it does not establish durable remission or long-term efficacy. The relapsing nature of HHD, the single-patient design, and the short follow-up after dose reduction require cautious interpretation. Longer follow-up and additional cases are needed to clarify whether reduced-frequency maintenance can be used reliably in selected patients.

## Data Availability

The original contributions presented in the study are included in the article/Supplementary Material. Further inquiries can be directed to the corresponding author.
